# Serum amyloid A and metabolic disease: evidence for a critical role in chronic inflammatory conditions

**DOI:** 10.3389/fcvm.2023.1197432

**Published:** 2023-06-15

**Authors:** Laura J. den Hartigh, Karolline S. May, Xue-Song Zhang, Alan Chait, Martin J. Blaser

**Affiliations:** ^1^ Department of Medicine, Division of Metabolism, Endocrinology, and Nutrition, University of Washington, Seattle, WA, United States; ^2^ Diabetes Institute, University of Washington, Seattle, WA, United States; ^3^ Center for Advanced Biotechnology and Medicine, Rutgers University, Piscataway, NJ, United States

**Keywords:** obesity, diabetes, cardiovascular disease, SAA, intestine, liver, adipocytes, macrophages

## Abstract

Serum amyloid A (SAA) subtypes 1–3 are well-described acute phase reactants that are elevated in acute inflammatory conditions such as infection, tissue injury, and trauma, while SAA4 is constitutively expressed. SAA subtypes also have been implicated as playing roles in chronic metabolic diseases including obesity, diabetes, and cardiovascular disease, and possibly in autoimmune diseases such as systemic lupus erythematosis, rheumatoid arthritis, and inflammatory bowel disease. Distinctions between the expression kinetics of SAA in acute inflammatory responses and chronic disease states suggest the potential for differentiating SAA functions. Although circulating SAA levels can rise up to 1,000-fold during an acute inflammatory event, elevations are more modest (∼5-fold) in chronic metabolic conditions. The majority of acute-phase SAA derives from the liver, while in chronic inflammatory conditions SAA also derives from adipose tissue, the intestine, and elsewhere. In this review, roles for SAA subtypes in chronic metabolic disease states are contrasted to current knowledge about acute phase SAA. Investigations show distinct differences between SAA expression and function in human and animal models of metabolic disease, as well as sexual dimorphism of SAA subtype responses.

## 1. Introduction

Members of the serum amyloid A (SAA) family are acute phase reactants and chemokines that are elevated in acute inflammatory conditions such as infection (1, 2), as well as chronic inflammatory conditions including autoimmune disorders (3–8), obesity (9–13), type 2 diabetes (T2D) (14, 15), and cardiovascular disease (CVD) (16–19) (reviewed extensively in 20, 21). Several SAA subtypes are present across diverse animal species (22), including invertebrates (23), suggesting important conserved functions. Since SAA is poorly soluble in aqueous solutions, it circulates associated with lipoproteins, in particular high density lipoprotein (HDL), and is considered an apolipoprotein (24, 25). Functions of particular SAA subtypes include roles in host defense (26–30), chemoattraction (31–34), lipid metabolism (35–37), and inflammation (38). We now review the emerging knowledge about distinctive functions of the different SAA subtypes.

### 1.1. SAA subtypes and receptors

Of the 4 known SAA subtypes, SAA1 and SAA2 are highly expressed in the liver in mammals including humans in response to inflammatory stimuli, and can circulate at high concentrations, usually bound to HDL (39). SAA1 and SAA2 are highly homologous, differing in only a few amino acids. In contrast, SAA3 is more highly expressed in extrahepatic tissues in particular animal species (40, 41). SAA3 is not known to circulate under most conditions, with the exception of high dose lipopolysaccharide (LPS) injection (42). SAA3 is considered to be a pseudogene in humans due to a premature stop codon (43), leading to a frame shift in codon 31, thereby deleting the last ten amino acids (44). SAA3 is only ∼40% homologous to SAA1/2. Since in humans SAA1 and SAA2 are expressed from both liver and extrahepatic tissues, it has been difficult to conclusively distinguish hepatic from extrahepatic SAA functions in humans. However, phenotypic distinctions between hepatic and extra-hepatic SAA subtypes in mice, due to the predominance of extrahepatic Saa3, allow sharper definition (18, 38, 44). SAA3 protein has been detected in human mammary gland epithelial cell lines (45), although its expression is more commonly found in non-human mammals. SAA4 is constitutively expressed by most cell types and responds only minimally to inflammatory stimuli (46, 47). In many prior studies, distinctions between specific SAA subtypes were not reported, perhaps due to the lack of available antibodies capable of distinguishing them. This is unfortunate, as it is possible that different SAA subtypes exert different functions in the context of metabolic disease. In this review, we use the term “SAA” to refer to SAA1/2, or to reflect that the authors of work described did not specify particular SAA subtypes. In addition, in accordance with scientific nomenclature standards, “SAA” will refer to humans, while “Saa” corresponds to mouse.

The major identified SAA receptors are listed in Table 1. SAA binds to formyl peptide like receptors 1 and 2 (FPLR1 and FPLR2) in human monocytes, neutrophils, human embryonic kidney (HEK293) cells, and human umbilical vein endothelial cells (HUVECs), thus promoting chemotaxis and increased calcium flux. In response to varied stimuli (Table 1), mitogen-activated protein kinases (MAPKs) and nuclear factor kappa B (NFκB) pathways are further activated, which leads to secretion of tumor necrosis factor alpha (TNFα), interleukin-8 (IL-8), and monocyte chemotactic protein-1 (MCP-1) (32, 33, 48–51, 69, 70). The receptor for advanced glycation end products (RAGE) is another known SAA receptor present on several tissues and cell types. SAA mediates the activation of the AGE/RAGE axis and NFκB pathways, with subsequent transcription of interleukin-6 (IL-6), heme oxygenase type-1 (HO-1) and monocyte colony stimulating factor (M-CSF) (52–55). Moreover, SAA induces signal transducer and activator of transcription 1 (STAT1)-mediated high mobility group box 1 (HMGB1) expression and protein kinase R (PKR) activation, potentially through RAGE and toll-like receptors (TLRs) (52). SAA has affinity for TLR2 and TLR4 (56–61, 71, 72), and to the oxidized low-density lipoprotein receptor (LOX-1) (62) and scavenger receptor class B type 1 (SRB1), thus mainly signaling via the MAPK pathway in both immune and epithelial cells. A recently described SAA receptor is Selenoprotein S/Tanis [SELS in humans (63, 65, 67, 68), Tanis in animal models (68)]. Tanis/SELS is highly expressed in liver, skeletal muscle, and adipose tissue (68), which may distinguish SAA effects mediated by this receptor from those found primarily on immune cells. SELS expression on adipose tissue is highly correlated to circulating SAA levels, suggesting a potential feed-forward mechanism (68, 73). Importantly, most of these potential SAA receptors respond to multiple ligands, with SELS having the highest degree of SAA-specificity. Collectively, varied SAA receptor expression patterns on different cell and tissue types could indicate different SAA functions.

**Table 1:** SAA receptors and downstream signaling pathways.

SAA receptors	SAA	Host specificity		Ligand(s)/stimuli	Target tissue/cell	Signaling pathways	Outcomes
		Human	Rodent				
FPRL1/ FPRL2	SAA1, SAA2	(32, 33, 48–51)	(32)	SAA (0.01–2 µM) rhSAA1 (20 µg) LPS (100 ng/ml) GM-CSF (100 ng/ml)	Neutrophils Monocytes Cell lines: HEK 293, HUVEC	Calcium signaling Cell migration NFκB MAPK (ERK/p38/JNK) AKT	↑ intracellular Ca^2+^ (32, 33, 51) ↑ chemotaxis (32, 33) ↑ IL-8, CCL2 (Cell media/ plasma) (50, 51) ↑ IL-8, TNFα mRNA/protein (48) ↑ p-ERK, p-p38, p-JNK (48, 50) ↑ p-AKT (48)
RAGE	SAA1, SAA2	(52, 53)	(52–55)	rSAA1 (0.1–10 µg/ml) LPS (5 mg/kg) AgNO_3_ (0.5 ml of a 2% solution) AEF (100 µg) Azocasein (7%) sRAGE VC1 (100 µg) peptide 5 (100 µg)	Kidney, Liver, Spleen Primary macrophages Cell lines: RAW264.7, THP-1, U937, BV-2	AGE-RAGE NFκB STAT1 PKR	↑ SAA (Tissue distribution) (55) ↑ NO (52) ↑ AGE, CML (plasma) (53) ↑ IL-6, IL-12, HGMB1, MCP-1, RANTES (Cell media/plasma) (52) ↑ RAGE, IL-6, HO-1, M-CSF mRNA (53, 54) ↑ p-STAT1, p-PKR (52)
TLR2	SAA1, SAA2	(56)	(56, 57)	rhSAA (1 µM) Concavalin (10 mg/kg)	Liver, Spleen Cell line: HeLa	TLR NFκB MAPK (ERK/p38/JNK)	↑ ALT, AST (57) ↑ SAA, SAF-1, IL-6, IFN_ϒ_, TNF-α (plasma) (56) ↑ SAA1, MCP-1, MIP1α/β, IL-1R, IL-10, IL-8, IL-18, IL-23, IP-10, eotaxin mRNA (56, 57) ↑ CD4+, Th17, T-reg, F4/80 + CD11b+ (57) ↓ p-IκBα (57) ↑ p-ERK1/2, p38, JNK (57)
TLR4	SAA1, SAA2, SAA3	_	(58–61)	SAA3 (0.3–1 µg/ml) LPS (0.01–1 µg/ml) Concavalin (10 µg) S100A8, S100A9 (70–100 µg)	Kidney, Liver, Lungs Primary macrophage Myeloid cells (Mac1+) Cell lines: MCF7, RAW264.7	NFκB MAPK (ERK/p38/JNK) AKT Rho GTPase	↑ Chemotaxis (58, 60) ↑ NO, iNOS (59) ↑ SAA3, IL-6, TNFα mRNA (58, 60) ↓ TLR4 mRNA (61) ↑p-IkB (58) ↑ p-ERK, p-p38, p-JNK (59, 60) ↑ p-AKT (59)
LOX-1	SAA1, SAA2, SAA3	_	(62)	hSAA3 (2 µg/ml) LPS (1 µg/ml)	Cell lines: LU65, LU99, MCF7, HUVEC H292, T47D	MAPK (ERK)	↔ hSAA2, hSAA3 mRNA (62) ↑ p-ERK (62) ↑ IL-6, IL-1β (plasma) (62)
SR-B1/CLA-1	SAA1, SAA2	(66, 67)	(63, 66)	SAA (0–10 µg/ml) Recombinant adenovirus SAA1/2 LPS (25 µg)	Cell lines: HeLa, HepG2, THP-1, CHO	Cholesterol efflux MAPK (ERK/p38)	↑ ABCA1- and SR-B1 dependent cholesterol efflux (64, 65) ↓ Cholesteryl ester uptake (63) ↑ p-ERK1/2, p-38 (63)
SELS/Tanis	SAA1, SAA2	(65, 68, 73)	(68)	Insulin (6–100 nM) Glucose (12–35 mM) Euglycemic-hyperinsulinemic clamp (40 mU·m^−2^·min^−1^)	Adipose tissue, skeletal muscle, liver Cell lines: HepG2, C2C12, 3T3-L1	MAPK (ERK/p38) Inflammatory pathways	↑ SAA (plasma) (68) ↑ IL-8 (Cell media) (66) ↑↓ Tanis/Sels mRNA (67, 68) ↑ p-ERK1/2, p-p38 (66) ↑ cardiometabolic risk factors (67, 68)

ABCA1, ATP-binding cassette A1; AEF, amyloid-enhancing factor; AGE, Advanced glycation end-products; AKT, protein kinase B; AEF, Amyloid-enhancing factor; ApoA1, apolipoprotein A1; CCL2, C-C Motif Chemokine Ligand 2; CHO, Chinese hamster ovary cells; CML, Carboxy methyl lysine; ERK, extracellular signal-regulated kinase; FPRL1, formyl peptide receptor-like 1; FPRL2, formyl peptide receptor-like 2; FPRL1, formyl peptide receptor-like 1; GM-CSF1, Granulocyte-macrophage colony-stimulating factor; HMGB1, high mobility group box 1 protein; HO-1, heme oxygenase 1; hSAA, human serum amyloid A; HUVEC, human umbilical vein endothelial cells; IκBα, nuclear factor of kappa light polypeptide gene enhancer in B-cells inhibitor alpha; IL-1β, interleukin 1 beta; IL-6, interleukin 6; IL-8, interleukin-8; IL-10, interleukin 10; IL12, interleukin 12; IP-10, Interferon gamma-induced protein 10; JNK, c-Jun N-terminal kinase; LOX-1, oxidized low-density lipoprotein receptor 1; MCP-1, monocyte chemotactic protein 1; M-CSF, macrophage colony-stimulating factor; MIP-1α, macrophage inflammatory protein 1 alpha; NFκB, nuclear factor kappa-light-chain-enhancer of activated B cells; NO, nitric oxide; PKR, protein kinase R; RAGE, receptor for advanced glycation end-products; RANTES, Regulated upon Activation, Normal T Cell Expressed and Presumably Secreted; rhSAA, recombinant human serum amyloid A; SAA1, serum amyloid A1; SAA2, serum amyloid A2; SAA3, serum amyloid A3; SAF-1, Serum amyloid A-activating factor-1; SELS, selenoprotein S; sRAGE, Soluble for advanced glycation end-products; SR-B1, scavenger receptor B1; TLR2, toll-like receptor 2; TLR4, toll-like receptor 4; TNFα, tumor necrosis factor-alpha.

↑, increased; ↓, decreased; ↔, non-affected.

### 1.2. SAA regulation in the acute phase response

Considerable research has been focused on the kinetics of hepatic SAA expression and secretion during an acute inflammatory response [reviewed in (20, 74)]. The mechanics of SAA expression and secretion vary with the stimulus type. Systemic levels of SAA can be 1,000-fold higher than baseline during an acute inflammatory response to sepsis (75, 76), viral infections including COVID-19 (1, 77, 78), vaccinations (79), or tissue trauma (80). The immediate systemic levels of SAA are primarily hepatic in origin during infection (44), with contributions from extra-hepatic sources following tissue trauma (81). Hepatic SAA production is triggered by bacterial products such as endotoxin or inflammatory cytokines interleukin-1 beta (IL-1β), interleukin-6 (IL-6), and TNFα that reach the liver (74). While much prior work has focused on the hepatic acute-phase SAA1 and SAA2 subtypes, important roles for extra-hepatic SAA in the chronic inflammatory processes associated with metabolic diseases are now emerging (82, 83).

### 1.3. SAA vs. CRP

Since its discovery nearly 100 years ago, C-reactive protein (CRP) has been used in clinical practice as a marker of acute inflammation (84). CRP is known to rapidly increase in response to infection or trauma, and has a short half-life that enables a rapid decrease when the stimulus ceases (85). However, SAA rises in parallel with CRP in the same acute inflammatory conditions, and may be a more sensitive marker for acute events (19, 86–88). Similar to CRP, hepatic SAA is regulated by the above inflammatory cytokines (IL-1β, IL-6, and TNFα 89, 90), although CRP can be induced by pathways related to interleukin-17 (IL-17) and hepatocyte nuclear factor (HNF), in contrast to SAA (91). In addition to inflammatory cytokines, hormones including glucocorticoids, leptin, and thyroid hormone also regulate SAA expression (92, 93). Indeed, SAA levels may be better predictors of coronary artery disease (CAD), cancer, and of related poor outcomes than CRP (19, 94). However, CRP levels more accurately predict poor outcome in elderly populations (95). SAA as a biomarker of acute infection or traumatic injury remains less widely used clinically due to a lack of robust calibration reagents and routine assays. There would be great value to developing reliable, robust, and cost-effective SAA clinical assays.

## 2. SAA in chronic metabolic diseases

**Figure 1:**
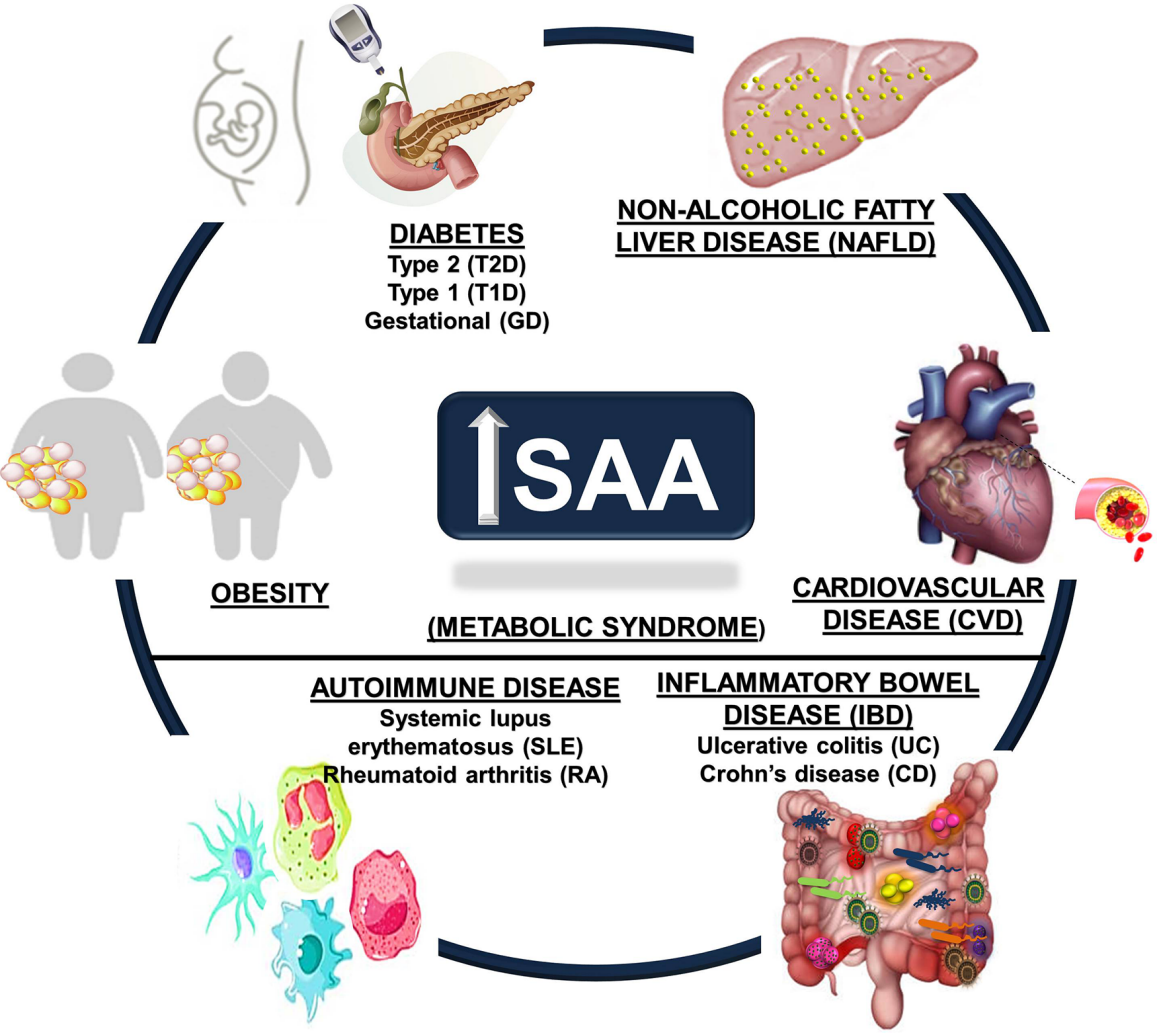
Metabolic disease states associated with increased circulating SAA. Obesity, cardiovascular disease (CVD), autoimmune diseases (including systemic lupus erythematosus (SLE) and rheumatoid arthritis (RA)), diabetes (Type 1, Type 2, and gestational), inflammatory bowel disease (IBD, including Crohn's disease (CD) and ulcerative colitis (UC)), and non-alcoholic fatty liver disease (NAFLD) are chronic metabolic conditions that are all associated with increased circulating SAA levels.

Chronic inflammatory conditions tend to promote much lower elevations in systemic SAA (∼3 to 10-fold) than acute inflammatory conditions and may be sustained, deriving from diverse tissues such as the liver, adipose tissue, lung, small and large intestines, and hematopoietic cells such as macrophages (9, 11, 18, 96–100). The markedly different systemic SAA levels observed in acute vs. chronic inflammatory conditions suggests the potential for different mechanisms (91), prompting speculation that SAA is an important concentration-dependent effector of innate and adaptive immune responses (44). Aging has been associated with increased SAA levels (83, 101, 102), as have aging-related metabolic conditions. Evidence for potential roles of SAA in several metabolic diseases are discussed in the sections that follow, with an emphasis on obesity, diabetes, non-alcoholic fatty liver disease (NAFLD), CVD, autoimmune conditions such as systemic lupus erythematosus (SLE) and rheumatoid arthritis (RA), and inflammatory bowel diseases (IBD) ulcerative colitis (UC) and Crohn's disease (CD) (Figure 1).

### 2.1. Obesity and metabolic syndrome

The increased circulating SAA levels observed in individuals with obesity are highly correlated with body mass index (BMI), body weight, adiposity, and SAA1 and SAA2 mRNA expression in white adipose tissue (WAT) (11, 96), and are not related to hepatic SAA1 or SAA2 expression (9, 97, 98, 103). Circulating SAA levels have been positively associated with visceral adiposity (104), suggesting visceral fat as a potential source. However, the relative contributions of subcutaneous and visceral WAT to SAA production are not known, nor has it been determined that WAT-derived SAA contributes to the circulating SAA pool in obesity (105), or whether WAT-derived SAA induces local cytokine production that stimulates hepatic SAA expression. SELS, a major SAA receptor, is expressed in adipose tissue and directly associates with adiposity and BMI (65), suggesting a potential feed-forward mechanism that contributes to the sustained adipose tissue inflammatory state in obesity (71). Whether increased SAA expression in WAT plays a local or systemic role in obesity pathogenesis, or whether it is merely a biomarker of disease severity, is unknown. The extent to which WAT, liver, or both contribute to systemic SAA levels has not been resolved.

An initial study of 34 subjects with obesity showed a 6-fold increase in SAA expression in subcutaneous WAT compared with 27 lean controls; this was associated with 20-fold higher expression from adipocytes than the WAT stromal vascular fraction (96), which contains pre-adipocytes, immune cells, and vasculature. A meta-analysis confirmed a strong positive association between BMI and circulating SAA levels (13), and showed that SAA1 and SAA2 expression was higher in subcutaneous WAT in people with overweight and/or obesity (97, 106). In addition, serum SAA levels are positively associated with adipocyte diameter (106, 107). Distinctions between SAA1 and SAA2 were generally not made in these early studies due to the lack of distinguishing primers and subtype-specific antibodies that persists.

Conversely, weight loss can reduce circulating and adipose tissue-derived SAA levels in humans. A meta-analysis of 10 studies showed that weight loss significantly reduced circulating SAA levels (13). Weight loss following a low-fat (LFD) (*n* = 19) or very low carbohydrate diet (VLCD) (*n* = 22) led to reduced circulating SAA levels proportional to the amount of weight lost and also associated with insulin resistance (88). Several independent studies showed that weight loss due to a VLCD in women (*n* = 33–48) was strongly associated with reduced plasma SAA and adipocyte-derived SAA (9, 10, 96, 108), while insulin sensitivity was not consistently affected (9, 108). These divergent phenotypes could reflect different subject characteristics, with postmenopausal women showing a metabolic benefit from SAA reduction (9, 10) while premenopausal women did not (108). Another study in 439 women reported similar reductions in plasma SAA with weight loss due to dietary intervention, but not exercise (109). Importantly, other inflammatory markers including MCP1 and CRP also decreased during weight loss (109). Roux-en-Y gastric bypass significantly reduced circulating SAA levels in women with obesity (*n* = 20) (110). Additional studies are required to determine whether specifically reducing SAA in the context of weight loss is beneficial.

Mouse studies parallel the observation that SAA levels are increased in humans with obesity, and that adipose tissue mRNA expression of Saa is similarly increased in the obese state. Initial studies identified Saa3 as the specific subtype expressed in murine adipocytes (111) and macrophages (112, 113), both essential for development of obesity. Ob/ob mice, which spontaneously develop obesity due to increased food consumption subsequent to leptin deficiency, have elevated circulating and adipose tissue Saa levels (114, 115). Further, diet-induced obese mice consistently have elevated Saa3 mRNA levels in adipose tissue (82, 116–121). However, obesity-associated adipose-derived Saa3 does not contribute to circulating Saa levels in mice (105). Mice engineered to express luciferase via the Saa3 promotor only show luciferase activity in adipose tissue following long-term high fat diet (HFD)-fed conditions, with no detectable luciferase in any tissue examined after one week of HFD or after acute injection with LPS, providing temporal data about Saa3 expression kinetics (121). However, using more sensitive mass spectrometry, we have shown that a single high dose LPS injection is sufficient to induce Saa3 expression in adipose tissue, associated with increases circulating Saa (42), an effect supported by identifying Saa3 in LPS-stimulated plasma using isoelectric focusing gels and ELISA (122).

Sleep deprivation has been associated with sharp increases in SAA. Circulating SAA levels increased by more than 4-fold in mice experiencing paradoxical sleep deprivation for 72 h, an effect coincident with increased adipose tissue Saa3 mRNA expression, but not Saa1/2 (123). Circulating Saa and Saa3 mRNA returned to basal levels when sleep was restored. Importantly, increased circulating SAA also has been observed in humans deprived of sleep for either 24 or 48 h (123). In another study, SAA levels were 2-fold elevated in 17 adults who regularly experienced obstructive sleep apnea, which disrupts sleep, compared to weight-matched controls (124). Obstructive sleep apnea is strongly associated with the metabolic syndrome (125), also associated with SAA levels, which may confound interpretation of these studies. Because sleep deprivation and disrupted sleep schedules increase risk for obesity and its complications, disrupted sleep-induced SAA could be considered a novel risk factor for metabolic disease.

Studies in which mouse Saa is perturbed genetically have yielded ambiguous results. Mice engineered to express human SAA1 from WAT had elevated circulating human SAA1 mirroring obesity levels even without an obesogenic stimulus (126), providing evidence that WAT-derived SAA circulates. However, overexpressing SAA1 from WAT had no observed effects on body weight, WAT inflammation, or glucose or insulin tolerance (127). Loss of extrahepatic Saa3 in obese mice led to improved local WAT inflammation and systemic lipoprotein profiles and to resistance to high fat diet (HFD)-induced obesity, particularly in female mice (82). By contrast, subsequent Saa3 knock out mice were more prone to HFD-induced obesity with increased adiposity (128). Further, triple knock-out mice (Saa1, Saa2, and Saa3-deficient) showed no effect of a HFD on body weight or adiposity, but had worsened glucose and insulin tolerance (129). These divergent results suggest that the distinct metabolic characteristics of the models used, such as the inclusion of dietary sucrose/cholesterol, which particular Saa subtypes are perturbed, or gut microbiota composition and function, could have major impacts on observed phenotypes related to Saa.

Despite such phenotypic differences in obesity when SAA subtypes were perturbed, several studies point towards SAA promoting adipose tissue expansion. Silencing Saa3 in cultured pre-adipocytes reduced their adipogenic potential, leading to smaller adipose tissue depots when injected into NUDE mice (130). Similarly, targeting Saa using anti-sense oligonucleotides reduced adipose tissue expansion and inflammation as well as circulating endotoxin levels in male Swiss Webster mice (131), suggesting that disrupting Saa signaling also improved intestinal barrier integrity. Increased Saa3 expression in visceral adipose tissue from obese mice is highly correlated with macrophage number and inflammatory expression profile (121), suggesting that interaction with macrophages may drive adipocyte Saa3 expression. Thus, the crosstalk between adipocytes and macrophages that promotes adipose tissue inflammation and subsequent insulin resistance in obesity may require SAA (121).

### 2.2. Type 2 diabetes and gestational diabetes

Excess visceral adiposity and increased systemic inflammation are associated with insulin resistance (132, 133), which is the reduced capacity for insulin-stimulated glucose uptake in metabolically active tissues such as adipose tissue and skeletal muscle. Pancreatic insulin secretion subsequently increases to compensate for reduced insulin sensitivity to maintain euglycemia. If the pancreatic beta cells are unable to secrete sufficient insulin to compensate for the reduced insulin sensitivity (termed beta cell dysfunction), hyperglycemia ensues, leading to glucose intolerance and eventually T2D (134). Cross-sectional studies in men of European, Asian Indian, and American descent have shown that total, visceral, and subcutaneous adiposity, BMI, and waist circumference are all negatively associated with insulin sensitivity (135, 136). In addition to its association with obesity, with a key contribution from adipose tissue, SAA is similarly associated with T2D in humans and in animal models. In 134 patients with T2D, circulating SAA levels strongly correlated with hemoglobin A1c (HbA1c) and homeostatic model assessment for insulin resistance (HOMA-IR) after controlling for age, sex, and BMI status (14), suggesting a relationship between SAA and insulin resistance.

In humans, diabetes and circulating SAA levels are strongly related (11, 107, 137–140), and a prospective association between SAA and incident T2D has been reported (15). In a study of 765 older men (mean age 77), 112 with T2D, serum SAA strongly correlated with diabetes status, an association lost when adjusted for BMI, waist circumference, or fasting insulin levels (141). In a small study, omental adipose tissue from subjects with diabetes (*n* = 6) had a 3-fold increase in SAA mRNA expression compared with non-diabetic controls (*n* = 10), and omental SAA expression strongly correlated with fasting glucose levels and total body fat mass (142). In 134 subjects with T2D, HbA1c and HOMA-IR strongly correlated with circulating SAA levels after controlling for age, sex, and BMI (14); the effect was reduced with adjustment for parameters related to glucose metabolism (15), suggesting linkage between SAA and insulin resistance. In subjects with both obesity and T2D, SAA is bound to apoB-containing lipoproteins including very low-density lipoproteins (VLDL) and low-density lipoproteins (LDL), in addition to HDL [its usual transport partner in plasma (37)], similar to observations in mice (143). The mechanism for SAA binding to these lipoproteins in people with diabetes is unknown. Evidence exists that a truncated form of SAA1, which is missing an N-terminal arginine, is reduced in subjects with T2D and is negatively associated with glycemic control (144). Adipose tissue SELS was positively associated with measures of glycemic control in both lean and obese subjects (65, 73), as well as in age- and weight-matched subjects with diabetes (145). Moreover, insulin increases SELS expression in cultured adipocytes (65), suggesting a potential feed-forward mechanism for increased SAA expression in insulin resistance. SAA disrupts insulin signaling in cultured adipocytes (120, 146), suggesting a potential mechanism for its association with T2D. Most T2D subjects also have abdominal obesity, making it difficult to tease apart obesity-specific and T2D-specific contributions of SAA.

However, a strong association exists between diabetes and SAA that is independent of obesity. One study of 182 T2D subjects showed elevated serum SAA levels compared to healthy weight-matched controls (*n* = 180), with mean BMI of 24 in both groups (147). A small study similarly showed that SAA levels were elevated in age- and weight-matched subjects with T2D compared with normoglycemic controls (73). Controlling for age, sex, and BMI revealed a sustained correlation between indices of glucose dysregulation (i.e., HbA1c, HOMA-IR) and SAA, suggesting an effect specific to the diabetic state (14). However, another study found no differences in SAA levels between weight-matched subjects with obesity or T2D (110). To our knowledge, only a single study has reported no differences in SAA between healthy insulin-sensitive subjects and those with T2D (148). Emerging evidence suggests that improving insulin sensitivity drives the reduction in SAA levels following weight loss. In a small study in which subjects with overweight or obesity were given rosiglitazone for 12 weeks, circulating SAA levels were reduced by 37% despite the absence of weight loss, and WAT explants from these subjects showed lower SAA secretion post-treatment (9). Pharmacotherapy for T2D (i.e., metformin, glipizide, rosiglitazone, insulin, or acarbose) reduces serum SAA levels in T2D subjects (9, 139, 149). Thus, while the diabetic state and SAA levels are directly associated, whether SAA plays a distinct role in T2D pathology independent of a role in obesity remains to be determined.

SAA levels are further elevated in subjects with T2D and nephropathy (147, 150) and retinopathy (151). SAA may be an important predictor for end-stage renal disease and death in patients with diabetic kidney disease, with elevated intra-renal SAA expression (152). SAA is elevated in T2D patients with proteinuria, with serum SAA levels positively associated with albumin excretion rate and glomerular membrane thickening (140, 153), consistent with a potential causal role.

Similar links between Saa and T2D have been observed in animal models. In mice, a HFD promotes early increases in Saa3 expression in white adipose tissue, with subsequently elevated hepatic levels of Saa1 and Saa2 (120). In these models, insulin resistance is highly correlated with circulating Saa levels (120). In hepatocytes, overexpression of the Saa receptor, Tanis, led to decreased insulin-stimulated glucose uptake and glycogen synthesis, indicating increased insulin resistance (73). Db/db mice, which lack the leptin receptor and spontaneously develop features resembling obesity and T2D, express high levels of Saa3 from adipocytes, but not the liver (114). In a common rodent model of T2D in which obesity is initiated by consumption of a HFD and hyperglycemia is triggered by the administration of low-dose streptozotocin (STZ), a beta cell toxin that promotes hyperglycemia, renal Saa3 is increased (154).

Systemic SAA levels are elevated in pregnancy, especially in women with gestational diabetes (GD) (101). Serum SAA levels were 14% higher in 39 pregnant women with GD than in 25 healthy controls, and SAA was positively associated with BMI, age, oral glucose tolerance test, and HbA1c levels (155). It is unknown whether GD itself increases systemic SAA levels, or whether increased SAA simply reflects gestational weight gain (156). While one study did not observe increased SAA levels in GD patients, decreased variability in SAA levels was observed (157). Further studies are required to conclusively determine if SAA plays a detrimental role in GD. Indeed, a prospective clinical trial (NCT04238936) aims to compare SAA levels between women diagnosed with GD and healthy controls.

### 2.3. Polycystic ovary syndrome (PCOS)

PCOS is a chronic inflammatory condition that impacts ∼5%–10% of women of reproductive age in industrialized countries and is associated with an increased incidence of obesity, diabetes, and atherosclerosis (158, 159). In a study of 83 subjects with PCOS, serum SAA levels were double those of 39 age-matched controls (160). Omental and subcutaneous WAT biopsies showed increased SAA mRNA and protein expression, suggesting that the circulating SAA derived at least in part from adipose tissue. Incubation of adipose tissue explants with glucose increased SAA production, providing evidence that SAA secretion may be regulated by hyperglycemia. PCOS subjects were insulin-resistant, and a 6-month treatment regimen with metformin reduced circulating SAA levels, suggesting a possible link between SAA and adipose tissue insulin sensitivity (160). Because PCOS is associated with enhanced WAT lipolysis (161), and WAT-derived SAA also augments lipolysis (9), we speculate that WAT-derived SAA may play a causal role in PCOS-mediated metabolic dysfunction.

### 2.4. Non-alcoholic fatty liver disease (NAFLD)

NAFLD is commonly present as part of the metabolic syndrome (162), a constellation of disorders that increase the risk for CVD and diabetes, including abdominal obesity, hyperglycemia/insulin resistance, hypertension, and dyslipidemia (163). NAFLD is characterized by triglyceride accumulation in hepatocytes (steatosis), which can progress to steatohepatitis, characterized by the accumulation of inflammatory cells. SAA levels often are elevated in patients with the metabolic syndrome (164, 165). SAA was found to be 2–3-fold higher in patients with non-alcoholic steatohepatitis relative to age-matched healthy controls (166). Because liver biopsy, the gold standard diagnostic test for the presence of NAFLD, is an invasive procedure, non-invasive biomarkers for this condition would be highly desirable. However, although SAA could potentially be a useful biomarker for NAFLD, it is too non-specific to justify its use for this purpose.

Mechanisms linking SAA and NAFLD remain speculative. In the Cohort on Diabetes and Atherosclerosis Maastricht (CODAM) study, in which alanine amino transferase (ALT) was used as a surrogate measure of NAFLD, multiple linear regression analysis was used to investigate the association between ALT and several metabolic syndrome components as potential mediators of the liver disease. Their findings suggest that insulin resistance is the key pathophysiological mechanism to explain the association between the metabolic syndrome and NAFLD, with adipose tissue inflammation, endothelial dysfunction and free fatty acid levels likely playing lesser roles (167). However, ALT is an imperfect biomarker for NAFLD. Cytokines produced by liver-resident and infiltrating inflammatory cells may play important roles in liver inflammation and NAFLD. SAA may exacerbate hepatic steatosis via the TLR4-mediated NFκB signaling pathway (168). Hepatocyte-derived SAA1 promotes intrahepatic platelet aggregation and aggravates liver inflammation in NAFLD (169). Studies using hypercholesterolemic mice deficient in IL-1α or IL-1β showed the importance of these two cytokines in transforming steatosis to steatohepatitis and liver fibrosis (170). Given the well-documented link between SAA and IL-1β, SAA may also be important for liver disease progression. However, this requires additional study.

### 2.5. Cardiovascular disease (CVD)

Inflammation is a hallmark of atherosclerosis (171), and a recent clinical trial, The Canakinumab Anti-Inflammatory Thrombosis Outcomes Study (CANTOS), for the first time showed in a proof-of-concept trial that inhibiting inflammation using an antibody against Il-1β decreased cardiovascular events (172). The relationship between inflammation and CVD has been extensively studied by measurement of the inflammatory marker, CRP, which consistently has been shown to be modestly and chronically elevated in CVD patients and to predict the risk of cardiovascular events in a similar manner to SAA (19, 173, 174), although SAA has not been studied as extensively as CRP. As noted earlier, acute phase SAA is a good predictor of coronary artery disease outcomes (19, 94).

SAA could simply be a biomarker of the chronic inflammatory state that is present in CVD, similar to CRP; alternatively, it may play pathogenic roles. As described below, considerable evidence points to its role as a mediator rather than simply being a marker of atherosclerotic CVD. In considering its possible mediating role, potential differences between effects of lipoprotein-bound SAA and free SAA derived from extrahepatic cells in the artery wall must be distinguished.

SAA mRNA is present in macrophages, smooth muscle cells and endothelial cells in human atherosclerotic lesions (18), findings that suggest an immune response within the atherosclerotic artery wall, in which locally generated SAA is unlikely to be associated with lipoproteins. However, other studies showed immunohistochemical colocalization of Saa with apolipoproteins, including apoA1, the major apolipoprotein of HDL, in murine atherosclerotic lesions (175), consistent with SAA being transported to the artery wall by plasma lipoproteins.

Several studies in mice provide evidence for Saa being an atherosclerosis mediator. LDL receptor (Ldlr)-deficient mice fed a pro-inflammatory diet with or without added cholesterol showed marked increases in plasma Saa levels, which correlated with atherosclerosis extent (116). Mice in which Saa was either overexpressed or silenced suggest Saa roles in atherosclerosis pathogenesis, although the data are not uniform. Chow-fed Apoe-deficient mice in whom Saa was overexpressed using a lentiviral vector had increased *en face* and aortic root lesions compared to control-fed mice, although differences were not observed with an atherogenic diet (176). Plasma levels of IL-6 and TNFα and expression of vascular cell adhesion molecule 1 (VCAM-1) and monocyte chemotactic protein-1 (MCP-1), and lesion macrophage content all increased with Saa overexpression (176). In another experimental approach, a single injection of a human Saa-containing adenovirus in Apoe-deficient mice increased plasma Saa levels for ∼10 days, leading to increased atherosclerosis (177). When repeated injections of the human SAA-containing adenovirus were administered to immune-deficient mice to prevent an antibody response to the human protein, brachiocephalic lesions and aortic lesion area were markedly increased (177). The authors postulated that the increase in atherosclerosis was due to SAA-mediated induction of transforming growth factor-β (TGFβ), which increased vascular biglycan expression and led to increased LDL retention (see later). Deficiency of Saa in Ldlr-deficient mice led to reduced atherosclerosis in the ascending aortic arch but not in the aortic root or innominate artery at 6 weeks, although this difference was lost by 12 weeks (178). Parallel findings were observed in male Ldlr-deficient mice also deficient in FPLR2, one of the major Saa receptors, although the effect was more prolonged than in the Saa/Ldlr double knockout mice (179). In both studies, transplantation of Saa-deficient bone marrow-derived cells replicated the findings, suggesting that the reduced atherosclerosis may have resulted from the absence of free Saa in lesions rather than in the circulation. However, in Apoe/Saa double knockout mice, no difference in lesion area was observed at ∼50 weeks (180), although no early time points were examined. A subsequent study in Apoe-deficient male mice also lacking Saa1 and Saa2 using Saa3 suppression with an anti-sense oligonucleotide showed significantly reduced atherosclerosis (181). These results imply that all acute phase Saa isoforms have pro-atherogenic properties, and that deficiency/suppression of all 3 acute phase isoforms is required for atheroprotection in mice. Saa3 effects on atherosclerosis were not reported in female mice, despite sexually dimorphic Saa3 expression (182) (see below). Saa transgenic rabbits failed to show an increase in atherosclerotic lesions (183). Therefore, in summary, while most mouse studies suggest that Saa contributes to the development of early atherosclerotic lesions, results in Saa-deficient models are not consistent, possibly related to the nature of the model and the timing of observations. Nevertheless, such studies raise the question of how Saa might affect the atherogenic process. Several potential mechanisms are plausible.

Since SAA can be expressed by several cells of the artery wall (18), including perivascular adipocytes (184) and macrophages (18, 112, 113, 182–187), the locally produced SAA in lesions unattached to lipoproteins could have signaling functions that might be atherogenic. These include activation of the NFkB and MAPK signaling pathways via interaction with receptors such as class B scavenger receptor CD36, TLR4, TLR2, FPLR2 and RAGE (176, 188, 189). Activation of monocytes/macrophages and perivascular adipocytes can generate chemoattractant molecules such as MCP-1, which could lead to the recruitment of additional inflammatory cells and vascular smooth muscle cells. SAA also can be a direct chemoattractant (190). Moreover, direct activation of the chemoattractant receptor, FPLR2, by free SAA could further attract inflammatory cells into developing vascular lesions. Free SAA also has been shown to induce a phenotypic switch in vascular smooth muscle cells towards a more proliferative type of cell that synthesizes more matrix molecules (188). However, *in vitro* studies using free SAA should be interpreted cautiously, since minor contamination with endotoxin could lead to similar effects.

HDL-bound SAA also may play a role in atherogenesis. When SAA is secreted by the liver as part of the acute or chronic inflammatory response, it circulates in plasma bound to HDL, although it can associate with less dense lipoproteins under certain circumstances (24, 25, 37, 143). HDL particles that carry SAA, so-called “inflammatory HDL”, is less atheroprotective than normal HDL, with reduced inhibition of inflammation in cells due to its being trapped by cell surface proteoglycans (191), versican in the case of adipocytes and biglycan produced by macrophages (118). Trapping of SAA-containing HDL at the cell surface prevents it from adequately promoting reverse cholesterol transport (192). HDL derived from inflamed mice devoid of Saa1 and Saa2 functioned normally, as it did when the proteoglycans were removed from the cell surface either chemically or by genetic manipulation (118). Humans treated with low levels of endotoxin also had impaired cholesterol efflux capacity from macrophages, despite no change in circulating HDL-cholesterol levels. Proteomic analyses showed that the cholesterol efflux capacity of HDL correlated inversely with Saa1 and Saa2 content (193). Binding of SAA-containing HDL by extracellular proteoglycans such as biglycan in humans (194) and perlecan in mice (174) may lead to HDL retention in the vascular intima, increasing susceptibility to oxidative and enzymatic damage similarly to trapped LDL (195). Retained HDL could thus be pro-atherogenic, compared to its more widely accepted anti-atherogenic properties. The products of oxidative and enzymatic damage to retained lipoproteins may play important roles in atherogenesis (196).

Finally, SAA might stimulate thrombosis, which often precipitates clinical events. SAA can induce tissue factor production by monocyte/macrophages (197) and platelet activation (198). Thus, SAA could play multiple roles in the atherosclerotic process from monocyte adhesion, inflammatory and smooth muscle cell chemotaxis, cellular inflammation, HDL function, retention of atherogenic lipoproteins in the artery wall, and thrombogenesis. The net effect is that SAA is likely to play a causative role in atherogenesis, although the extensive data are not fully consistent.

### 2.6. Type 1 diabetes

In contrast to T2D, type 1 diabetes (T1D) develops as a result of autoimmune destruction of pancreatic beta cells, reducing insulin production capacity; subjects with T1D thus require exogenous insulin to maintain euglycemia. Little is known regarding SAA and T1D. One study has shown that SAA levels were elevated in 1,139 subjects with T1D compared with 848 healthy controls (199); however, these plasma donors were not age-matched, and the T1D subjects tended to be older. However, SAA increased specifically in HDL in subjects with T1D compared to age-, sex-, and BMI-matched controls, an effect much stronger when subjects were stratified by HbA1c and was not observed for CRP (200). A common T1D model can be generated in mice by injecting them with the pancreatic beta cell toxin STZ, leading to beta cell apoptosis (201, 202). In such STZ-treated mice, circulating SAA levels increased (203), with increased Saa3 expression specifically from adipocytes (114). Whether hyperglycemia or STZ itself stimulated adipose Saa3 was not determined. However, treating cultured 3T3-L1 adipocytes with 12–25 mM glucose induces Saa3 mRNA expression (114, 190, 204), an effect replicated by hyperglycemic clamps in mice (114), suggesting that hyperglycemia is the critical factor. Whether glucose-stimulated SAA expression changes systemic SAA levels or performs local functions is not known. As with studies related to T2M, mechanistic studies are needed in mice to determine whether SAA is sufficient or required for the pathology of T1D.

### 2.7. Autoimmune diseases: systemic lupus erythematosus (SLE) and rheumatoid arthritis (RA)

SLE and RA are chronic diseases in which a person's immune system attacks its own tissues, resulting in inflammation and tissue damage in affected organs. While RA can be physically debilitating but typically is not life-threatening, SLE can lead to severe complications such as kidney failure, seizures, and increased risk of thrombosis. SAA may be a biomarker for both conditions (3, 6, 205–207). SAA promotes T-helper 17 (Th17) differentiation (208, 209), which plays important immunologic roles. However, excessive Th17 responses also can promote autoimmune conditions including SLE and RA (210). In patients with RA, their joints contain elevated SAA (6, 205), with levels correlating with plasma SAA levels and disease progression (211, 212). Rather than simply diffusing into joints from the bloodstream, SAA itself may be expressed in synoviocytes, macrophages, and endothelial cells within synovial tissues in RA patients (6, 213). Computational modeling identified Saa3 as the gene most strongly correlated with the severity of collagen-induced arthritis (214). Moreover, synovial fibroblasts isolated and cultured from patients with RA produced 2–4 times more SAA than those from healthy subjects (213). Whether SAA directly contributes to these autoimmune diseases remains to be elucidated. A potential mechanism is that in RA patients, SAA and associated cytokines potently induce matrix degrading enzymes in synovial fibroblasts (213, 215, 216), which if left unchecked could contribute to disease pathogenesis and joint destruction. Treatment with the TNF antagonist etanercept reduces RA disease severity while simultaneously reducing circulating SAA levels (217), providing one linkage between SAA and RA, but the causal direction is unknown.

### 2.8. Inflammatory bowel disease (IBD)

The two major types of IBD include ulcerative colitis (UC) and Crohn's disease (CD). Both are complex conditions that result from chronic dysregulated immune function in the gastrointestinal tract (218). UC is limited to the colon; however, CD can involve any part of the gastrointestinal tract, but usually affects the distal small intestine and/or the colon (219). Previous work suggests that SAA may be a more sensitive biomarker for IBD than CRP (8, 220, 221), as SAA levels remain elevated while CRP disappears in patients who are in clinical remission (222).

Patients presenting with either UC or CD consistently show elevated serum SAA levels (220, 223). In humans, intestinal biopsies from CD patients showed significantly increased colonic SAA1/2 expression levels (224). From an extensive panel of inflammatory markers, including CRP, IL-22, and IL-6, SAA had among the highest positive associations with a Simplified Endoscopy Score for CD (SES-CD, *r* = 0.4), fecal calprotectin (*r* = 0.39), Crohn's Disease Activity Index (CDAI, *r* = 0.14), and stool frequency (*r* = 0.18) (223). Such studies link intestinal SAA to potential roles in disease development or protection. Subjects with CD without mucosal healing had higher SAA levels than subjects in clinical remission (225), suggesting SAA as a marker for CD severity. In patients with UC who were in remission, consumption of a low-fat, high-fiber diet improved quality of life, in conjunction with reduced circulating SAA levels (226). In one clinical trial, SAA was a highly significant predictor of CD severity, and treatment with filgotinib, a selective JAK1/STAT inhibitor, improved CD symptoms while simultaneously reducing circulating SAA levels (223).

Mouse models of IBD similarly display elevated circulating SAA levels. Systemic SAA as well as local Saa3 expression levels become elevated within days of administration of dextran sodium sulfate (DSS) in drinking water in a mouse model of colitis (227–229), an effect that may function to protect colonic epithelium from acute injury by recruiting IL-22-producing neutrophils (228). This does not appear to be specific to that model, as mice given trinitrobenzone sulfonic acid (TNBS) via colonic catheter, in another well-studied colitis model, also responded with increased systemic SAA (230, 231). Pharmacological treatments including 6-thioguanine and cyclosporine A, utilized to improve colitis outcomes in mice, effectively reduced circulating SAA levels (229), as did administration of *Bacillus subtilis* spores as a probiotic (230). To date, only a few studies have indirectly examined IBD phenotypes with concurrent SAA genetic perturbation. One study showed that mice concurrently deficient in Saa1, Saa2, and Saa3 had attenuated colitis as assessed by histology (209). However, in the proximal colon of the mouse, Saa1 and Saa2 expression is confined to the epithelium, while Saa3 expression is found in immune cells including monocytes, macrophages, and dendritic cells (209). These data suggest that Saa1/2 exert system-wide functions of mucosal sensing and defense, while Saa3 drives local function, due in part to the differential potentiation of Th17 responses by these subtypes (209). Similarly, mice that were deficient in Saa1 and Saa2 that had colitis-associated colon cancer showed attenuated weight loss, gut histological damage, and gut inflammation (232), findings that suggest that Saa1/2 may augment colitis severity. Saa1/2 deficiency resulted in reduced Saa3 colonic expression (232). Conversely, specific deletion of only Saa3 rendered mice more susceptible to dextran sulfate sodium (DSS)-induced colitis (228), implying that Saa3 may be protective against IBD. Collectively, the precise roles for different SAA subtypes in IBD remain unknown, but emerging evidence suggests that Saa1/2 and Saa3 have different triggers and functions.

## 3. Tissue- and stimulus-specific SAA effects

Expression kinetics for each SAA subtype varies greatly by tissue source and stimulus type. While SAA1 and SAA2 are primary players in the acute phase response, in mice Saa3 may play a more prominent role in local inflammation. Mice express Saa3 in many extra-hepatic tissues including adipose tissue, lung, macrophages, and small/large intestine, with the liver predominantly producing Saa1 and Saa2 (40). This distinct division in murine subtype expression patterns enables the study of extra-hepatic Saa in metabolic disease. Extra-hepatic Saa expression appears predominant in chronic inflammatory conditions, while Saa derives largely from the liver in more acute inflammation (9, 18, 20, 91, 96, 98, 100). In humans, it is much more difficult to separate the contribution of extra-hepatic SAA to metabolic disease phenotypes in humans, because SAA1 and SAA2 are expressed both from liver and extra-hepatic tissues. Thus, much of our knowledge of extra-hepatic SAA originates from mouse models. In this section, the various SAA subtypes and their expression patterns in response to particular stimuli from various tissue and cell types will be discussed (Table 2).

**Table 2:** Tissue- and stimulus-specific SAA effects in humans and rodents.

Tissue/cell type	Model	Stimulus	SAA subtypes^a^
Liver			
Human	HepG2 cells (234) HepG2 cells (89, 248) HepG2 cells (252) Primary hepatocytes (251) Liver tissue (98)	IL-1β, IL-6 TNFα (10 ng/ml) IL-6 TNFα, DEX IL-1 β LPS (20–500 ng/ml) Obesity	↑↑ SAA1, SAA2 (15–25-fold) ↔ SAA1, SAA2 ↑↑ SAA1, SAA2 (5-fold) ↔ SAA1, SAA2 ↑ SAA1 ↑↑ SAA1, SAA2 ↔ SAA1
Rodent	BALB/c mice (40, 238) Swiss mice (236) C57Bl6/J mice (122, 233) Primary hepatocytes (122) Mouse fibrosis model (239) Mouse HSCs (239) C57Bl6/J mice (42) Ldlr-/-.Leiden mice (244) FVB mice (114) db/db mice (114) C57Bl6/J (121) Mouse liver (113) Mink liver (269) C57Bl6/J (209)	LPS (50 µg) Casein (0.5 ml 10%) LPS (100 µg) LPS (100 µg) LPS (25 µg) Ccl4 (0.5 µl/g) Ccl4 (0.5 µl/g) LPS (25 µg) AgNO_3_ (0.5 ml 1%) Casein (0.5 ml 5%) DIO (50 weeks HFD) STZ- hyperglycemia Genetic T2D DIO (16 weeks) Amyloidosis LPS (3 mg/kg) MOG- autoimmune encephalomyelitis	↑↑ Saa1, Saa2, Saa3 (100-fold) ↔ Saa1, Saa2 ↑↑ Saa1 ↑↑ Saa1, Saa2, Saa3 (2,000–5,000-fold) ↑↑ Saa1, Saa2, Saa3 ↑ Saa1, Saa3 (10–40-fold) ↔ Saa1, ↑ Saa3 (40-fold) ↑↑ Saa1–4 (200, 2,000, 1,000-fold) ↑↑ Saa1, Saa2 (400, 10,000-fold) ↔ Saa4, ↑ Saa1–3 (60, 1,000, 8-fold) ↔ Saa1 ↔ Saa3 ↔ Saa3 ↔ Saa3 ↓ Saa1, Saa2 ↑ Saa1 ↑↑ Saa1–2, ↔ Saa3 (10, 20-fold)
Adipocytes/WAT			
Human	Omental and SQ adipose tissue (9) SQ adipose tissue (96, 98) MADS (253) Primary breast adipocytes (254)	Obesity DEX, insulin Rosiglitazone Obesity Obesity → weight loss rSAA (1–30 µg/ml) DHA (50–100 µM)	↑↑ SAA1 ↑ SAA1 (6-fold secretion) ↓ SAA1 (70% reduction in secretion) ↑ SAA1–2, SAA4 (6-fold) ↓ SAA1, SAA2 (1.6–2.2-fold) ↑ SAA1 (7-fold) ↑ SAA1
Rodent	C57Bl6/J mice (42) C57Bl6/J mice (122) Ldlr-/-.Leiden mice (44) 3T3-L1 adipocytes (114) FVB mice (114) ob/ob mice (114, 121) db/db mice (114, 121) 3T3-L1 adipocytes (190) 3T3-L1 adipocytes (245) C57Bl6/J (121) 3T3-L1 adipocytes (246) 3T3-L1 adipocytes (242) Swiss Webster mice (271)	LPS (25 µg/mouse) AgNO_3_ (0.5 ml 1%) Casein (0.5 ml 5%) LPS (100 µg) DIO (50 weeks HFD) TNFα, LPS Insulin, Rosi, IL-6 Hyperglycemia (25 mM) LPS (100 ng/g) STZ- hyperglycemia Obesity Genetic T2D SFA (12:0, 14:0, 16:0) Hyperglycemia (25 mM) PUFA (20:4, 20:5, 22:6) IL-1β DIO (16 weeks) LPS LPS + RAW264.7 cells rSAA (5 µg/ml) Presense of microbes	↑↑ Saa1, Saa2, Saa3 (60, 30, 750-fold) ↔ Saa1–3 ↔ Saa1–3 ↑ Saa1 (100-fold), ↑↑ Saa3 (400-fold) ↓ Saa1 ↑↑ Saa3 ↔ Saa3 ↑ Saa3 ↑↑ Saa3 (200-fold) ↑ Saa3 ↑↑ Saa3 ↑ Saa3 (8-fold) ↑ Saa3 (2-fold) ↑ Saa3 (5-fold) ↓ Saa3 ↑ Saa3 (10,000-fold) ↑ Saa3 (11-fold) ↑ Saa1 (2-fold) ↑ Saa1 (2-fold) ↑ Saa3 (3-fold) ↑ Saa3 (10-fold), ↔ Saa1, Saa2
Monocytes			
Human	PBMC (250) THP-1 cells (187) U-937 cells (187) HL-60 cells (187)	IL-1β, IL-6, TNFα LPS + DEX, or DEX LPS, DEX, IL-1, IL-6 LPS, DEX, IL-1, IL-6	↑ SAA2 (7-fold) ↑ SAA1 ↔ SAA1 ↔ SAA1
Macrophages			
Human	Coronary artery sections (18) THP-1 macrophages (187) U-937 macrophages (187) HL-60 macrophages (187)	Atherosclerosis LPS DEX DEX LPS, DEX, IL-1, IL-6	↑ SAA1 ↔ SAA1 ↑ SAA1 ↑ SAA1 ↔ SAA1
Rodent	BALB/c mice (40) RAW264.7 cells (246) RAW264.7 cells (271) Kupffer cells (233) Peritoneal macrophages (233) Peritoneal macrophages (113) Microglia, MDM (209)	LPS (50 µg) LPS LPS LPS LPS Amyloidosis MOG- autoimmune encephalomyelitis	↑↑ Saa3 (100-fold) ↔ Saa1 ↑ Saa3 ↑ Saa1 ↑ Saa1 ↑↑ Saa3 ↑ Saa3
Intestine			
Rodent	BALB/c mice (40) C57Bl6/J (121) CONV-R vs. GF mice (271) CONV-R vs. GF mice (278) CMT-93 colonic epithelial cells (224, 271) Mink intestine (269)	LPS (50 µg) Casein (0.5 ml 10%) DIO (16 weeks) Presence of microbes Presence of microbes LPS LPS (3 mg/kg)	↑↑ Saa1 (1–4-fold), Saa3 (3–25-fold) ↔ Saa1, Saa3 ↔ Saa3 ↔ Saa1, Saa2, ↑ Saa3 ↑↑ Saa1, Saa2, Saa3 (4-fold) ↑↑ Saa3 (7-fold), ↔ Saa1–2 ↑↑ Saa1

12:0, lauric acid; 14:0, myristic acid; 16:0, palmitic acid; 20:4, arachidonic acid; 20:5, eicosapentaenoic acid; 22:6, docosahexaenoic acid; AgNO_3_, silver nitrate; Ccl4, tetrachloride; CLA, conjugated linoleic acid; CONV-R, conventionally-reared; DEXv dexamethasone; db/db mice: DIO, diet-induced obesity; leptin receptor-deficient mice; DEX, dexamethasone; DHA, docohexaenoic acid; GF, germ-free; HepG2, human hepatoma cells; HSC, hepatic stellate cells; IL-1β, interleukin 1 beta; IL-6, interleukin 6; LPS, lipopolysaccharide; MDM, monocyte-derived macrophage; MOG, myelin oligodendrocyte glycoprotein; ob/ob mice, leptin-deficient mice; PBMCs, peripheral blood mononuclear cells; PUFA, polyunsaturated fatty acids; rSAA, recombinant SAA; SFA, short chain fatty acids; SQ, subcutaneous; STZ, streptozotocin; T2D, type 2 diabetes; TNFα, tumor necrosis factor alpha; WAT, white adipose tissue; ↑, modest increase; ↑↑, robust increase; ↔, no change.

^a^ Fold-mRNA expression, unless otherwise indicated.

### 3.1. Liver

The liver is perhaps the most frequently studied SAA-expressing tissue, wherein hepatic resident macrophages (i.e., Kupffer cells) produce Saa3 (in mice) and hepatocytes make SAA1/2 (233, 239). As such, an influx of immune cells could specifically increase Saa3 expression in the liver in mice. In cultured hepatocytes, particular combinations of cytokines predictably increase SAA1 and SAA2 gene and protein expression (234). Hepatocytes secrete high levels of SAA during an acute inflammatory insult in mice and humans (22). HepG2 cells, a human hepatocyte cell line, can express SAA1 and SAA2 in response to IL-1β and IL-6 in a dose-dependent manner, an effect augmented by pre-treatment with dexamethasone or TNFα (89, 248, 250). Primary human Kupffer cells co-cultured with hepatocytes secrete high levels of SAA2 following treatment with IL-1β and IL-6 (237), suggesting a potential paracrine signaling mechanism.

As discussed above, potent inflammatory stimuli initiate robust, rapid, but short-lived (∼24 h) SAA1 and SAA2 expression from the liver. LPS at dosages ranging from 0.25 to 100 µg/mouse increases murine hepatic mRNA expression of Saa1 (up to 2,000-fold), Saa2 (up to 200-fold), and to a lesser extent Saa3 (up to 40-fold) in an NFκB-dependent manner, with circulating SAA levels subsequently increasing to 3,000 µg/ml (42, 236, 238). All three SAA subtypes reach peak hepatic mRNA expression 12 h after LPS administration (238). Only high-dose LPS (25 µg) increases circulating Saa3 in mice (42, 122). Similarly, LPS activates SAA1 and SAA2 mRNA expression and secretion in human primary hepatocytes (251). Patients with sepsis have elevated SAA levels (235), which are stronger predictive markers of sepsis severity (76). SAA was a more sensitive and earlier predictor of neonatal sepsis than the more traditional CRP (75).

Other models of sterile inflammation in mice also have been shown to increase hepatic Saa levels. Silver nitrate (AgNO_3_), administered by subcutaneous injection of 0.5 ml of a 1% solution, increases hepatic Saa1 (40-fold), Saa2 (1,000-fold), and Saa3 (200-fold), and leads to circulating Saa levels equivalent to that observed with high doses of LPS (42). However, in contrast to findings after LPS, we did not find evidence of Saa3 in plasma following AgNO_3_ injection (42). Injection with casein (administered by subcutaneous injection of 0.5 ml of a 5% solution) modestly increased hepatic Saa1 (6-fold), Saa2 (100-fold), and Saa3 (5-fold) mRNA expression, resulting in much smaller increases in plasma Saa levels (42). Collectively, acute inflammatory stimuli differ in the resulting hepatic expression levels of SAA1–3, leading to varied systemic SAA concentrations, suggesting differential regulation.

In metabolic disease states such as obesity and T2D, hepatic SAA expression likely results from cytokine signaling from extra-hepatic tissues such as WAT (240, 252). A recent study identified SAA1 protein from both WAT and liver as a candidate biomarker associated with low-grade inflammation. There was a much stronger correlation of SAA1 with inflammation in the liver than with WAT inflammation (244), suggesting a more dominant hepatic role of SAA1. However, this particular study mined gene ontology datasets using general inflammatory search terms, so the particular metabolic conditions (i.e., obesity) of the original study subjects were not indicated.

### 3.2. Adipocytes

The acute inflammatory studies cited above showed effects on SAA subtypes expressed in the liver, but there also were strong SAA responses in adipose tissue. While reported hepatic SAA responses to LPS in mice are largely due to Saa1 and Saa2, adipose tissue responds to LPS with massive (∼500-fold) increases in Saa3 mRNA compared with 40-fold Saa3 increases in the liver (42). This effect appears to be LPS-specific, as neither AgNO_3_ or casein altered Saa1, Saa2, or Saa3 mRNA levels in adipose tissue (42). Thus, we speculate that LPS can induce expression of all three Saa subtypes in both liver and adipose tissue that all contribute to circulating levels, while AgNO_3_ and casein primarily target hepatic Saa. In this section, we present evidence for differential SAA subtype expression in response to several inflammatory mediators and metabolic factors.

Many stimuli have been shown to increase Saa3 mRNA and protein expression in cultured adipocytes. These include high levels of glucose (114, 190, 204), saturated fatty acids (190, 204), conjugated linoleic acids (204), pro-inflammatory cytokines including TNFα and IL-1β (114, 190, 245), and LPS (114). Conversely, anti-inflammatory stimuli such as polyunsaturated fatty acids (190) and rosiglitazone (114) reduce adipocyte Saa3 expression. In addition to chemical activation, 3T3-L1 adipocytes also express Saa3 in response to macrophage-derived components (121, 246), suggesting an important role in cell-cell communication, with similar effects observed in cultured human adipocytes and in mice. Human SGBS cells treated with saturated fatty acids display increased Saa1 expression, while polyunsaturated fatty acids decreased glucose-induced Saa1 (190), suggesting that the major adipose SAA subtype in humans is SAA1. Mice injected with LPS robustly increased Saa3 expression in visceral WAT comparable to hepatic Saa1/2 expression levels in the same mice (42); using mass spectrometry methods, Saa3 was identified in their plasma (42), suggesting that Saa3 can circulate under particular inflammatory conditions.

Recombinant (i.e., exogenous) SAA can directly impact adipocyte metabolism. In cultured 3T3-L1 adipocytes, recombinant SAA (rSAA, 5 µg/ml) reduced adipogenesis, accompanied by reduced adipogenic transcription factors and proteins including peroxisome proliferator-activated receptor gamma (PPARγ), CCAAT enhancer binding protein beta (C/EBPβ), and GLUT4 (242). rSAA also reduced lipid accumulation, increased lipolysis, prevented glucose uptake, triggered secretion of inflammatory cytokines IL6 and TNFα and increased mRNA expression of Saa3. In multipotent adipose-derived stem (MADS) cells isolated from human subcutaneous adipose tissue induced to differentiate into primary adipocytes *in vitro*, free- and HDL-associated rSAA increased MCP-1, IL-6, and IL-8 secretion in a dose-dependent manner (253). This pro-inflammatory phenotype was dependent on NFkB, not due to endotoxin contamination (243, 253). Moreover, rSAA treatment reduced mRNA expression of adiponectin, fatty acid synthase (FAS), C/EBPα, PPARγ, and GLUT4 (253, 254), suggesting impaired adipogenesis capacity. A propensity for rSAA to increase lipolysis also has been reported in human adipose tissue (9). The pro-inflammatory, pro-lipolytic, and anti-adipogenic effects of SAA also have been shown in primary porcine adipocytes (243).

Recent technical advances have enabled the study of adipose tissue down to the single-cell level (255, 256). Spatial transcriptomics on human subcutaneous abdominal adipose tissue sections has revealed 3 distinct subsets of adipocytes, including those rich in genes for leptin (Adipo*^LEP^*), the lipid droplet-associated proteins perilipin1 and −4 (Adipo*^PLIN^*), and SAA1/2 (Adipo*^SAA^*) (257). Adipo*^LEP^* was enriched in genes encoding matrix metabolism, Adipo*^PLIN^* in genes associated with lipid and glucose metabolism, and Adipo*^SAA^* in multiple retinol-binding adipokines (i.e., RBP4) (257). These have been linked with obesity co-morbidities including T2D, hepatic steatosis, inflammation, and metabolic syndrome (258, 259). Approximately 8% of the adipocytes examined were Adipo*^SAA^*, with similar proportions in donors with or without obesity, but there was high variability among donors (from 2%–18% of all adipocyte populations) (257). Whether or not the proportion of Adipo*^SAA^* cells differs in omental WAT, or is related to sex, is of interest.

In addition to secreting adipokines and nutrients into the circulation, adipocytes also secrete extracellular vesicles (EVs), including microvesicles, exosomes, and apoptotic bodies (260). EVs are heterogeneous membrane vesicles secreted by many cell types, including adipocytes, and function to facilitate intercellular communication within and between tissues via protein signaling, immune responses, and nutrient transport (261). EVs contain diverse cargo including proteins, lipids, and miRNAs. Adipocyte-derived exosomes can be identified by their adipocyte-specific protein cargo, chiefly adiponectin and perilipin (262). EVs differing in cellular origins possess unique biological properties, enabling cell- or tissue-specific effects. EV production derived from WAT is increased during obesity (263–266), and is correlated with insulin resistance in both humans and in animal models. SAA1 and SAA2 have been identified in EVs isolated from human adipose tissue (262), and Saa3 observed within vesicle-like structures within murine adipose tissue (121). These findings raise the possibility that adipose tissue-derived SAA communicates systemically with other target tissues, in addition to its local effects.

### 3.3. Macrophages

Macrophages are present in all peripheral tissues and contribute to systemic metabolism. Macrophage classification schema are emerging, but largely revolve around their functional potential, including the capacity to elicit an inflammatory response and ability to phagocytose pathogens and cellular debris (267). As such, macrophages can either contribute to or resolve inflammation. Moreover, macrophages that only reside within particular tissues often receive their own classification, such as hepatic Kupffer cells or central microglia. All tissues from which Saa3 expression can be detected have a dynamic macrophage population, suggesting a potential common source of Saa3.

In obesity, adipose tissue exhibits both increased SAA expression (SAA1 and SAA2 in humans and Saa3 in mice), as well as increased macrophage infiltration. Importantly, all SAA subtypes are expressed from macrophages (187). Initial studies showed that acute inflammatory stimuli, including LPS and casein, induced only Saa3 mRNA in murine macrophages (40, 113). Saa3 mRNA also increases in activated RAW264.7 macrophages (121), murine bone marrow-derived macrophages (121), murine J774.1 macrophages (18, 112), and murine foam cells within atherosclerotic lesions (18), but not in the human THP-1 cell line (187). Saa3 protein co-localizes with F4/80^+^ macrophages in obese adipose tissue (121).

That macrophages express SAA subtypes as well as SAA receptors, including TLR2, TLR4, RAGE, and SRB1, suggests autocrine activities that likely contribute to local effects (20, 268). Deletion of putative SAA receptors yields a blunted macrophage response to SAA. BMDMs from mice deficient in TLR2 exhibit a blunted inflammatory response to SAA (1 µM) (56), and neutralizing antibodies to TLR2 blunted SAA-mediated activation of THP-1 macrophages (20). Similar effects have been observed in peritoneal macrophages from TLR4-deficient mice (59). SAAs may bind to macrophage-produced extracellular matrix (ECM) components, including proteoglycans and glycoproteins (195). Collectively, an increasing body of work connects SAA and macrophages.

Monocytes freshly isolated from humans or monocytic cell lines consistently respond to SAA with potent pro-inflammatory responses. Within an hour of treating with rSAA, peripheral human blood mononuclear cells (PBMCs), THP-1 monocytic cells, and monocyte-derived macrophages (MDMs) all exhibit rapid expression of IL-1β, MCP1, IL-6, IL-8, TNFα, and macrophage inflammatory protein 1 alpha (MIP-1α), an effect that is sustained for 8–24 h and is similar to LPS (241). Similar effects were observed in RAW264 monocytes treated with rSAA, which yielded a pro-inflammatory phenotype characterized by increased MCP-1, IL-6, IL-8, and TNFα secretion (9). While a potent inflammatory stimulus (i.e., LPS or casein) initiates a robust, rapid, but short-lived (∼24 h) hepatic Saa response, and from macrophages directly treated in culture, a similarly rapid but more prolonged Saa3 response (72 h) has been observed in isolated peripheral macrophages, indicating markedly different hepatic expression kinetics (40, 113). Whether such different expression kinetics reflect a more prolonged response that is cell-type specific or is an effect secondary to the acute phase response remains to be determined.

### 3.4. Intestine

Intestinal SAA can be induced by several mechanisms, which are complicated by the potential for differing SAA subtype expression from varying intestinal cells. SAA1/2 are highly expressed in intestinal epithelium and in the endothelium lining the intestinal submucosal blood vessels in rabbits, rodents, and humans (224, 269, 270). Conversely, Saa3 has been detected at low levels in mouse colonic epithelium (224), but is more prevalent in intestinal immune cells (209). Moreover, in mice, Saa3 expression is more strongly induced by LPS and microbes in colonic epithelium than Saa1/2 (224, 247, 271). Induction of SAA1 and SAA2 in small intestinal epithelial cells by commensal microbes requires both IL-23 and IL-22 in a STAT3-dependent manner (272). Male Syrian hamsters injected with LPS (100 µg/g body weight) also expressed high Saa levels (unknown subtypes) in the duodenum, jejunum, and ileum (273). Mouse intestinal Saa3 is most closely related to human SAA1 with 70% amino acid homology (271), and may serve local gut functions (247).

SAA expression differs markedly throughout the intestinal tract, with SAA2 having the most variable expression between the ileum and rectum in subjects with IBD (274). Germ-free mice have very low ileal levels of Saa1 and Saa2, but higher expression in the colon than in conventional mice (275). These findings are consistent with an anti-bacterial SAA role in relation to an omnipresent colonic microbiota, and a much more variable ileal microbiota. As conventional mice mature, intestinal Saa rises in the ileum, reflecting the increasing bacterial load, but do not change in the colon. Perturbing early-in-life gut microbiome affected intestinal Saa expression. With pulsed therapeutic-level antibiotic (PAT) exposures at early ages after weaning, non-obese diabetic (NOD) mice have consistently decreased Saa1/2 and Saa3 expression in the ileum but not in the colon (249, 276, 277). Younger mice (P12) had significantly increased Saa1/2 and Saa3 expression in both ileum and colon two days after antibiotic exposure ended, indicating that intestinal Saa can biphasically respond to gut microbiome changes in patterns that are both age- and microbiome context-dependent during this critical period for host immune development. The early-life antibiotic-exposed mice showed significantly increased Saa1/2 and Saa3 expression in the ileum but not in the colon at P17 days (277). These studies further confirmed that early-life intestinal SAA expression is subject to regulation linked to gut microbiota composition, potentially reflecting an ancient evolutionary strategy to regulate the establishment of immune responses or tolerance in the developing animal.

Mono-colonization of germ-free mice with segmented filamentous bacteria (SFB) rapidly induces expression of Saa1, Saa2, and Saa3 in the terminal ileum, consistent with the unique spatial expression patterns of SAA in the gut. Induction of ileal Saa is further increased by conventionalization using fecal microbial transplant (FMT) from specific pathogen-free (SPF) mice (278). Induction of ileal Saa1 and Saa2 by SFB is mediated through the IL-23/IL-22 circuit in ileal epithelial cells (272). The SFB-induced ileal Saa proteins promote Th17 cell differentiation from ileal lamina propria dendritic cells and contribute to protective immune responses in the ileal mucosa (278).

Conversely, as anti-bacterial molecules, SAA may modulate gut bacterial growth and composition either directly or through downstream intestinal immune responses. Consistent with observations in mice, *in vitro* studies showed that overexpression of Saa1/2 in intestinal epithelial cell lines reduces growth of co-cultured bacterial cells (224). Similarly, in zebrafish SAA in intestinal epithelial cells derived via transgene expression constrains the bactericidal activity of neutrophils, and promotes neutrophil recruitment to the intestine that is functionally distinct from hepatic SAA expression (279). In a mouse model of DSS-induced colitis, Saa induction in the large intestine was required to dampen local inflammation, while SAA1/2 overexpression in cultured epithelial cells reduced the viability of co-cultured *E.coli (*224), suggesting a potential bactericidal function of SAA that may contribute to barrier integrity. Transgenic mice engineered to overexpress Saa1 are partially protected against inflammatory responses to cecal ligation and puncture (280), suggesting an inverse relationship between gut-derived SAA and inflammation.

The anti-inflammatory properties of intestinal Saa1 are most specific to LPS-induced inflammation, an effect that could be dosage-dependent. Saa1 has the ability to bind LPS and form a complex, which then facilitates the clearance of LPS by macrophages (280). The transition of Saa1 from exerting pro-inflammatory effects to anti-inflammatory effects may reflect the proteolysis of the Saa1 protein. The N-terminal and C-terminal domains of Saa1 are crucial for its pro-inflammatory activity, and their removal via proteolysis can transform Saa1 into an anti-inflammatory agent (280, 281). Whether other SAA proteins also are capable of switching from pro-inflammatory to anti-inflammatory functions is unknown. The precise functions of intestinal SAAs deserve further investigation.

## 4. Sexual dimorphism of SAA

Circulating SAA is positively associated with BMI and adiposity, with a propensity to also associate with fasting glucose, insulin, HbA1C, and HOMA-IR. An emerging literature describes unique sexual dimorphic relationships between SAA and several metabolic disease states. Fully characterizing sex differences in SAA expression kinetics and functional potential is thus of great importance.

Large-scale RNA-sequencing studies of healthy humans showed that adipose tissue contains ∼3,000 sexually differentiated genes, one of the highest levels of all tissues examined (282). There was higher expression in women of all known SAA subtypes (SAA1, SAA2, and SAA3(p) (the SAA3 pseudogene)), which were among the most highly sex-differential genes (283). In contrast, with the exception of breast and skin, no other SAA-expressing tissues (i.e., liver, lung, blood) show SAA subtypes in their lists of sex-biased genes (283). These findings have been replicated in several large-scale sequencing studies spanning dozens of tissues in healthy men and women (284, 285), and in mice (286). SELS, a major SAA receptor, is elevated in the adipose tissue of subjects with T2DM and correlated with measures of glycemic control (73), but sex was not investigated in these studies. Collectively, many studies indicate that adipose tissue from female mice and humans expresses higher SAA than tissue from males, but the involvement of sex differences in the pathophysiology of obesity or associated metabolic disorders is not known.

Healthy women (with BMI < 25) have higher circulating SAA than age-matched men, despite the men having a slightly higher average BMI (287). SAA positively correlates with BMI, waist circumference, waist-to-hip ratio, insulin, and HOMA-IR in both sexes. After adjusting for BMI, only the correlations with insulin and HOMA-IR remained significant for men, but not women.

One of the first studies to address potential sex differences in SAA kinetics characterized the association between adipocyte size and circulating SAA levels in men and women over a large range in BMIs, with the additional aim to examine potential associations with measures of glycemic control (107). Women generally had higher circulating SAA levels than men, and stronger correlations with BMI, adiposity, subcutaneous adipocyte diameter, fasting insulin, HOMA-IR, and leptin (107). This could relate to the higher proportion of subcutaneous WAT in women than men.

By contrast, the liver, the source of most acute-phase SAA, has rarely been implicated in sex differential SAA expression. In contrast to several studies that have not found SAA to be differentially expressed by sex in the liver (282, 283), one study has shown that males tend to express slightly higher levels of SAA subtypes than female mice. Male CD-1 mice have modestly higher hepatic mRNA expression levels of Saa1, Saa2, and Saa3 than females (288). Adipose tissue-derived SAA may be impacted by sex steroids, as WAT is highly enriched in these molecules (289), with levels widely varying in metabolic disease. In experiments using cultured murine peritoneal macrophages and BMDMs, testosterone and 17β-estradiol directly impacted Saa3 gene expression (182). Saa3-deleted macrophages show sexually dimorphic responses to sex steroids. After estradiol exposure, Saa3-deficient BMDMs harvested from male mice showed a massive increase in inflammatory gene expression compared to wild-type macrophages, with concurrent elevation of the estrogen receptor (182). Thus, a relationship between macrophages, sex steroid signaling, SAA, and metabolic disease is present but needs further definition.

Our prior studies have supported a potential sexual dimorphic role of Saa3 in a mouse model with global Saa3 deficiency (82). When given a high fat high sucrose (HFHS) diet, female mice, but not male mice, were protected from body weight gain and associated insulin resistance. To determine whether there was similar sexually dimorphic protection against atherosclerosis in female mice, we crossed our global Saa3-KO mice with mice deficient in LDLR, which promotes hypercholesterolemia and is a common model for studying atherosclerosis. In that study, male Saa3^−/−^ Ldlr^−/−^ mice were protected from atherosclerosis, while female Saa3^−/−^ Ldlr^−/−^ mice were not (182). We speculate that in these models, Saa3 modulates effects via pathways that could be tissue-specific. In the obese state, Saa3 is expressed primarily from hypertrophic adipocytes, and also expressed from adipose tissue macrophages (96). Conversely, in the setting of hypercholesterolemic atherosclerosis, Saa3 expression likely originates from aortic and/or hepatic macrophages in addition to adipose tissue. Thus, in these different models, Saa3 deficiency leads to divergent phenotypes in males and females.

Other studies also suggest a potential interaction between sex hormones and SAA. Women with RA had higher SAA levels than men with RA (211, 212, 290). In a linear regression model involving the ratio of estradiol to testosterone (E2:T), sex and the E2:T ratio were highly significant and independent predictors of circulating SAA (290). Women with BMI < 25 have also been reported to have higher SAA levels than men (287), and SAA correlates more strongly with BMI and adiposity in women than in men (11). These observations suggest that sex hormones play roles in regulating SAA expression. Circulating SAA is higher in women taking oral estrogen-containing contraceptives (291, 292) and in women undergoing estrogen replacement therapy (287, 293). The apparent estradiol-mediated increase in SAA observed in these studies was secondary to elevations in CRP. More work is required to determine the mechanisms linking sex hormones and SAA.

Phenotypic responses to pro-inflammatory stimuli have differed in macrophages harvested from male or female mice (182). Compared to male mice, bone marrow-derived macrophages (BMDMs) isolated from female mice and treated with pro-inflammatory fatty acids or LPS showed lower levels of inflammatory cytokine expression (294). This effect appears to be cell-autonomous, since sex hormones were not present. Transplanting male bone marrow into donor female mice led to a phenotypically male pattern of obesity-associated adipose tissue inflammation (294). However, the absence of Saa3 in BMDMs negated this inherent sex-specific effect (182). The specific interactions between Saa3 and sex hormones remains to be characterized, but could explain the sexually dimorphic observations related to SAA expression in metabolic disease.

## 5. SAA-targeting therapies

Targeting SAA may be a potential therapeutic avenue for dampening inflammation. One approach is to target pathways that will reduce SAA expression. Tocilizumab, a monoclonal antibody that targets IL-6 and reduces SAA levels (295), has been effective in treating a small number of patients with amyloidosis involving the gastrointestinal tract (296) and kidneys associated with Familial Mediterranean Fever (297, 298), and amyloidosis associated with rheumatoid arthritis (299), but this approach could potentially also be developed for use in other chronic inflammatory conditions. Anakinra and canakinumab, monoclonal antibodies that target IL-1β, have been used to reduce SAA levels in inflammatory conditions such as Familial Mediterranean Fever (300) and gouty arthritis (301). Moreover, the CANTOS trial, for the first time, showed that inhibition of inflammation using an antibody against Il-1β decreased cardiovascular events (172), providing further evidence for the importance of inflammation in atherosclerosis. Since SAA appears to play a role in the pathogenesis of atherosclerosis (see previous sections), it is possible an approach that inhibits Il-1β could be more widely adapted for preventing atherosclerosis, as well as rheumatic diseases and even in hyperinflammatory states associated with COVID-19 (302).

SAA contains binding sites that are specific for heparin and heparin sulfate, which have been postulated to be useful for preventing amyloidogenic conformation of SAA (303). SAA also inhibits acyl coenzyme A cholesterol acyltransferase and enhances cholesterol esterase activities shifting stored intracellular cholesteryl esters to free cholesterol, which can be transported from cells. Liposomal preparations of small synthetic peptides of SAA can bind and neutralize SAA, facilitating reverse cholesterol transport and preventing and reversing aortic lesions in mouse models of atherosclerosis (304). Eprodisate, which binds to the glycosaminoglycan binding site on amyloid fibrils, thus preventing polymerization and tissue deposition, may slow the progression of AA amyloidosis-related renal disease (64, 305), and also may be applicable to other amyloid related conditions.

All these approaches are still in experimental phases, but demonstrate potential proof-of-concept mechanisms for future SAA-targeted therapies.

## 6. Concluding remarks and perspectives

Elevations of SAA subtypes have been consistently associated with metabolic diseases such as obesity, diabetes, CVD, and autoimmune conditions in humans and in animal models. After 40 years of investigation, evidence is not yet sufficient to determine whether SAA plays causal roles in metabolic disease development and progression, or is merely a biomarker of broader phenomena akin to CRP. In this review, we have presented evidence that associations with several metabolic disease states differ in expression kinetics and dominant SAA subtypes, as well as tissue, cellular, and spatial expression patterns, implicating the tissue microenvironment as crucial to SAA function. In particular, while evidence suggests that WAT SAA expression increases in obesity, whether such increases contribute to the circulating SAA pool is not known. Due to distinct subtype expression patterns in mice vs. humans, it could be possible for WAT-SAA to circulate in humans, but not in mice. As such, we propose that the SAA functions associated with metabolic disease are physiologically distinct from those in acute-phase reactions. Moreover, accumulating evidence suggests that different SAA subtypes, long considered to be pro-inflammatory molecules, may play beneficial roles in conditions like IBD, highlighting the importance of the microenvironment for particular SAA-mediated phenotypes. Finally, we speculate that SAA could play important roles in the differential progression of sexually dimorphic metabolic conditions.
